# Micron-Sized Monodisperse Particle LiNi_0.6_Co_0.2_Mn_0.2_O_2_ Derived by Oxalate Solvothermal Process Combined with Calcination as Cathode Material for Lithium-Ion Batteries

**DOI:** 10.3390/ma14102576

**Published:** 2021-05-15

**Authors:** Zhuo Chen, Fangya Guo, Youxiang Zhang

**Affiliations:** College of Chemistry and Molecular Sciences, Wuhan University, Wuhan 430072, China; chenzhuo0005@whu.edu.cn (Z.C.); guofangya@whu.edu.cn (F.G.)

**Keywords:** lithium-ion batteries, Ni-rich cathode materials, LiNi_0.6_Co_0.2_Mn_0.2_O_2_, solvothermal method, micron-sized monodisperse particle, sintered temperature, electrochemical properties

## Abstract

Ni-rich cathode LiNi_x_Co_y_Mn_1-x-y_O_2_ (NCM, x ≥ 0.5) materials are promising cathodes for lithium-ion batteries due to their high energy density and low cost. However, several issues, such as their complex preparation and electrochemical instability have hindered their commercial application. Herein, a simple solvothermal method combined with calcination was employed to synthesize LiNi_0.6_Co_0.2_Mn_0.2_O_2_ with micron-sized monodisperse particles, and the influence of the sintering temperature on the structures, morphologies, and electrochemical properties was investigated. The material sintered at 800 °C formed micron-sized particles with monodisperse characteristics, and a well-order layered structure. When charged–discharged in the voltage range of 2.8–4.3 V, it delivered an initial discharge capacity of 175.5 mAh g^−1^ with a Coulombic efficiency of 80.3% at 0.1 C, and a superior discharge capacity of 135.4 mAh g^−1^ with a capacity retention of 84.4% after 100 cycles at 1 C. The reliable electrochemical performance is probably attributable to the micron-sized monodisperse particles, which ensured stable crystal structure and fewer side reactions. This work is expected to provide a facile approach to preparing monodisperse particles of different scales, and improve the performance of Ni-rich NCM or other cathode materials for lithium-ion batteries.

## 1. Introduction

Rechargeable lithium-ion batteries (LIBs), which have a high energy density and long cycle life, have a wide range of applications in sustainable and renewable technologies, including portable electronic devices and electric vehicles (EVs), thus encouraging the research into LIBs [1,2,3,4]. Cathode materials with a high energy density are crucial to the performance of LIBs because the conventional anode (graphite) can transfer a higher specific capacity (372 mAh g^−1^) [5]. Commercial cathode materials include LiCoO_2_ and LiFePO_4_. LiCoO_2_ has a higher cost and level of toxicity due to its Co content. The conductivity of LiFePO_4_ is limited due to its olivine structures. Compared with these commercial cathode materials, the Ni-rich layered oxides LiNi_x_Co_y_Mn_1-x-y_O_2_ (NCM, x ≥ 0.5) indicate a promising future for LIBs due to their higher reversible capacities, thermal stability, and lower cost [6,7,8].

The family of Ni-rich NCMs mainly consists of different ratio transition metals (TM), which can alleviate a number of problems, including the unstable structures and high costs of LiCoO_2_, LiNiO_2_, and LiMn_2_O_4_ in synergistic combinations with transition metals, while maintaining a number of positive properties. High Ni content provides relatively high discharge capacity, and is caused by Ni^2+^/Ni^3+^ and Ni^3+^/Ni^4+^ redox reactions. Reliable rate capability is maintained due to Co^3+^/Co^4+^ redox reactions caused by the slight Co content. The presence of Mn^4+^ assures thermal and structural stability [9,10,11]. Traditional commercial Ni-rich NCMs are spherical secondary particles (micron-sized) composed of many randomly oriented primary particles (nano-sized). However, extensive applications of traditional Ni-rich NCMs are restricted due to their inferior structural stability and poor cycle stability, which, in turn, results from (1) a larger specific surface area caused by the gap between primary particles, which increases the number of side reactions or structural corrosions of the electrolyte and (2) a portion of microcracks is easily generated because of the random orientation of primary particles during the Li^+^ extraction/insertion process, resulting in the pulverization of secondary particles—this is a key reason for capacity loss during the cycle [12,13,14,15]. Several strategies, including optimizing synthesis parameters [16,17,18], coating [19,20,21], and element doping [22,23,24], have been devoted to address these issues and enhance the properties of NCMs. In addition, another more effective approach to improve electrochemical performance is to fabricate micron-sized monodisperse particles [25,26,27]. Monodisperse NCM particles are composed of single crystals rather than an agglomeration of many nanocrystals. Therefore, the micron-sized monodisperse particles can decrease the specific surface area and mitigate the formation of microcracks. These features are beneficial for electrochemical performance in LIBs [28,29,30,31].

Several methods are used to prepare NCMs with micron-sized monodisperse particles, such as the co-precipitation, sol–gel, and molten salt methods [32,33,34,35]. Qian et al. [35] synthesized micron-sized single-crystal LiNi_0.6_Co_0.2_Mn_0.2_O_2_ via an industrially-applicable molten salt method, the material showed a high capacity of 183 mAh g^−1^ at 0.1 C, and it was confirmed that the particles were stable against microcracks under normal operating conditions. Duan et al. [25] reported on LiNi_0.8_Co_0.15_Al_0.05_O_2_ crystals with a monodisperse micrometer-scale particle distribution using the co-precipitation method, the material showed an initial discharge capacity of 174.5 mAh g^−1^ at 1 C and 91.7% capacity retention after 100 cycles. Liang et al. [26] investigated the mechanisms regarding the influence of three factors on the morphology and electrochemical properties of micron-sized single primary particle LiNi_0.8_Co_0.1_Mn_0.1_O_2_ materials using the molten salt method. However, these methods each have relatively complicated processes, e.g., the co-precipitation method provides micron-sized spherical particles with uniform mixing and high density, but requires the complex control of parameters and results in significant ammonia pollution; and the sol–gel method can produce a mixing process at an atomic level, resulting in the uniform composition and high purity of the materials, but the longer preparation period limits the synthesis efficiency [36]. The hydrothermal/solvothermal method is also called the one-pot method due to its simple synthesis path in an autoclave. Moreover, high vapor pressure decreases the activation energy of TM oxide materials, which is beneficial for the crystal growth process [37,38,39,40]. Therefore, the desire for easier operation supports the use of the hydrothermal/solvothermal method to maintain competitiveness in preparing excellent Ni-rich NCM materials.

In this study, from the perspective of convenience, micron-sized particles with monodisperse characteristics of Ni-rich LiNi_0.6_Co_0.2_Mn_0.2_O_2_ (NCM622) cathode materials were synthesized using a solvothermal method combined with calcination at high temperatures. The oxalate precursor, Ni_0.75_Co_0.25_C_2_O_4_ ∙2H_2_O, was first obtained during the solvothermal step. The oxidation of Mn^2+^ to Mn^3+^ or Mn^4+^ was reduced during an extended solvothermal reaction, and the crystal structure was stabilized. The Mn source was mixed into the precursor during the subsequent calcination process [41]. The influence of sintering temperature on the structure, morphology, and electrochemical properties of NCM622 was investigated systematically. The obtained materials had a micron-sized dispersed primary particle morphology at a suitable sintering temperature. Due to the monodisperse characteristics, the material exhibited reliable electrochemical performance, and provides a new means to prepare cathode materials with enhanced performance.

## 2. Materials and Methods

### 2.1. Material Preparation

**Scheme 1:**
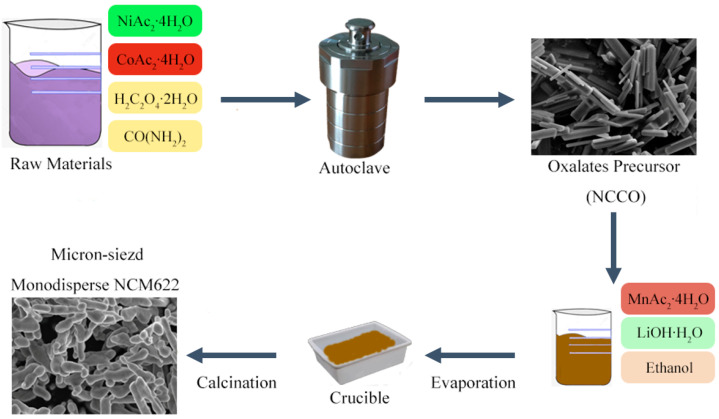
Schematic diagram of the preparation route for micron-sized monodisperse NCM622.

Scheme 1 shows a schematic of the preparation route for NCM622. The oxalate precursor, Ni_0.75_Co_0.25_C_2_O_4_∙2H_2_O (NCCO), was synthesized using the solvothermal method. Stoichiometric amounts of Ni(CH_3_COO)_2_∙4H_2_O, and Co(CH_3_COO)_2_∙4H_2_O (total 10 mmol) were dissolved in a mixed solution of deionized water and ethanol (1:1 *v*/*v*). Urea was added to the solution at a molar mass of 0, 10, and 20 mmol (i.e., the molar ratio of urea/(Ni + Co) = 0, 1, 2), the corresponding as-prepared samples were named NCCO0, NCCO1, and NCCO2, respectively. After dissolving in 10 mmol H_2_C_2_O_4_∙2H_2_O, blue homogeneous solutions were obtained by stirring. Then, the mixtures were transferred into an autoclave and annealed at 180 °C for 12 h to collect precipitates through centrifugation, which were washed and dried after cooling to room temperature. The NCM622 calcined precursor was obtained using a wet mixing procedure. Typical experiments were as follows: 2.4 mmol NCCO, 0.6 mmol Mn(CH_3_COO)_2_∙4H_2_O, and 4.5 mmol LiOH∙H_2_O (i.e., Li/TM = 1.5) were dispersed in ethanol, and brown powders were gained after evaporation of the ethanol. The precursors were pre-sintered at 500 °C for 6 h to decompose impurities and they were then calcined at different temperatures (750 °C (NCM750), 800 °C (NCM800), and 850 °C (NCM850)) for 12 h under an oxygen flow. NCM622 was obtained after cooling to room temperature.

### 2.2. Material Characterization

The crystal structures were identified using X-ray powder diffraction (XRD, Bruker, D8 Advance, Karlsruhe, Germany) with Cu Kα radiation (λ= 1.5406 Å) in the 2θ range of 10° to 80°. The morphologies were observed by field effect scanning electron microscopy (acceleration voltage: 5 kV, FESEM, Zeiss, SIGMA, Oberkochen, Germany) and high-resolution transmission electron microscopy (acceleration voltage: 200 kV, HRTEM, JEOL Ltd., JEM-2010FEF, Tokyo, Japan), and the surface elemental distribution of particles was investigated by electron dispersive X-ray spectroscopy (EDX, Oxford Instruments, Ultim Max 40, Oxford, UK). The chemical state of elements on the surface was examined by X-ray photoelectron spectroscopy (XPS, Thermo Fisher Scientific, Escalab 250Xi, Waltham, MA, USA).

### 2.3. Electrochemical Characterization

Electrochemical performance was examined using CR2016 coin cells with lithium metal disks as the counter electrodes. The working electrodes were fabricated by coating a mixed slurry of active materials, acetylene black, and polyvinylidene fluoride (PVDF) dissolved in N-Methyl-2-pyrrolidone (NMP) with a weight ratio of 80:15:5 on Al foils, which were used as the current collectors. The electrolyte consisted of 1 M LiPF_6_ in ethylene carbonate (EC)/dimethyl carbonate (DMC) (1:1 *v*/*v*) solvents. The separators were microporous films (Celgard, 2300, Charlotte, NC, USA). The cells were assembled in an Ar-filled glove box. Electrochemical tests were performed galvanostatically at different current densities in a voltage window of 2.8–4.3 V using the battery test system (Neware, CT-4008T, Shenzhen, China) at room temperature. Cyclic voltammograms (CV) were determined at 0.1 mV s^−1^ between 2.8 and 4.3 V. Electrochemical impedance spectroscopies (EIS) were calculated in a frequency range from 100 kHz to 0.01 Hz with an AC amplitude of 5 mV. Both CV and EIS were measured using the electrochemistry workstation (Shanghai CH instruments, CHI760C, Shanghai, China).

## 3. Results and Discussion

**Figure 1:**
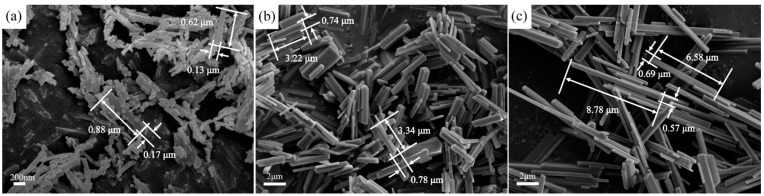
SEM images of NCCO precursor samples: (**a**) NCCO0, (**b**) NCCO1, and (**c**) NCCO2.

Figure S1 shows the XRD patterns of the NCCO precursor samples. All the samples can be indexed by their characteristics based on hydrated NiC_2_O_4_∙2H_2_O and CoC_2_O_4_∙2H_2_O, indicating a complete reaction between the metal acetates and oxalic acid using the solvothermal method [42]. The NCCO morphology with rod-like shapes is shown in Figure 1; the length of NCCO particles increased with the rising ratio of urea. The urea can release NH^4+^ in solvent under heating, forming [Ni(NH_3_)_n_]^2+^ or [Co(NH_3_)_n_]^2+^ complexes, which can inhibit crystal nucleation and promote the growth of crystal, resulting in different morphologies of oxalate precursors [38]. Therefore, NCCO1 was chosen as a precursor to synthesize NCM622 due to its uniform particle distribution.

**Figure 2:**
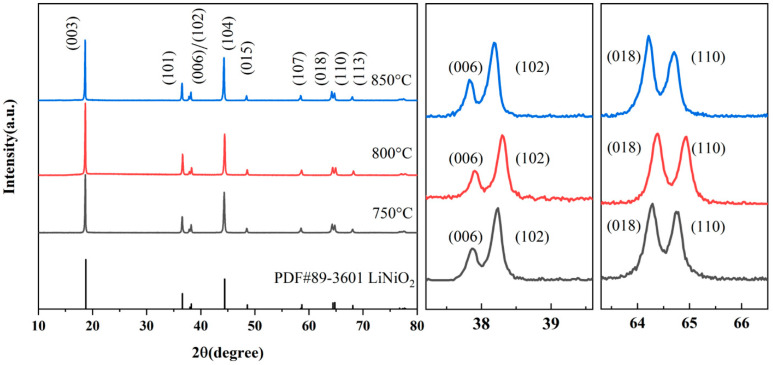
XRD patterns of NCM622 samples with different sintering temperatures.

The XRD patterns of the NCM622 samples with different sintering temperatures are shown in Figure 2. All of the peaks are well indexed to a hexagonal α-NaFeO_2_ structure (space group: R-3m), and no impurities can be observed in the patterns, which proves that NCM ternary materials were obtained after calcination. The NCM structure quality and lattice parameters are shown in Table 1. All of the samples display a value of c/a over 4.899, combined with clear peak splits of (006)/(102) and (018)/(110), which is a sign of a well-ordered layered hexagonal structure. Owing to the similar ionic radii of Li^+^ (0.69 Å) and Ni^2+^ (0.76 Å), the Li^+^ sites in the Li layer are easily occupied by the Ni^2+^ in the TM layer, and the increase in “cation mixing”, which results in a lower Li^+^ diffusivity because of the blockage caused by the Ni^2+^ in the Li layer. Therefore, Li^+^ conductivity decreases as the degree of mixing ratio increases. The cation mixing degree between Li^+^ and Ni^2+^ is reflected in the intensity ratio of I(003)/I(104), with a higher ratio corresponding to lower cation mixing [23]. The samples of NCM750, NCM800, and NCM850 presented I(003)/I(104) values of 1.61, 1.75, and 1.41, respectively, implying that NCM800 had the minimum degree of cation mixing. 

**Figure 3:**
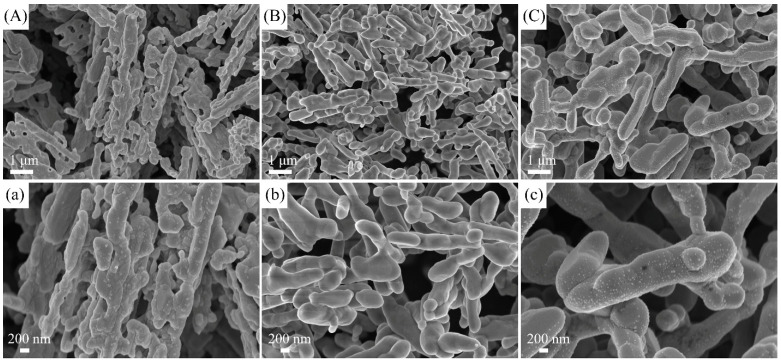
SEM images of (**A**,**a**) NCM750, (**B**,**b**) NCM800, and (**C**,**c**) NCM850.

Figure 3 shows SEM images of NCM622 with different sintering temperatures. Unlike the traditional spherical NCMs formed by agglomeration of nano-sized primary particles, the morphologies of NCM622 are relatively independent primary particles with a micron sized, and the size and dispersion of the particles increased with the increase in sintering temperature. Some uneven shapes were inevitably generated after calcination, but the NCM800 particles showed relatively few irregular particles and were well dispersed with a length of 2–3 μm, showing a certain degree of monodisperse particle characteristics. In contrast, in addition to the presence of more irregular particles in NCM750 and NCM850, several fine particles were attached on their surfaces, which was attributed to the inhomogeneous solid-phase reactions caused by unsuitable sintering temperatures [43], and the smoothest surface morphology can be observed for NCM800. Therefore, the sintering temperature is a significant factor for crystal growth and material morphology. A sintering temperature of 800 °C is appropriate for the formation of particles with relatively more monodisperse characteristics and for a greater number of uniform solid-phase reactions between precursors and lithium salts.

**Figure 4:**
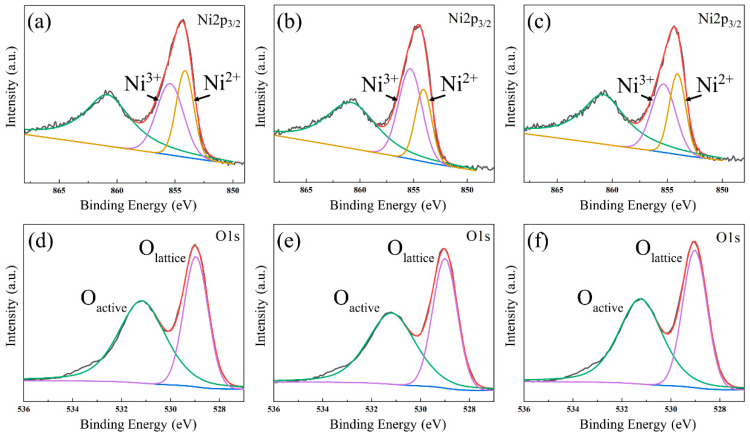
XPS spectra of Ni2p and O1s (**a**,**d**) NCM750, (**b**,**e**) NCM800, and (**c**,**f**) NCM850.

The valance states of NCM622 with different sintering temperatures were analyzed using XPS. The spectra of Ni2p and O1s are shown in Figure 4. As shown in Figure 4a–c, the Ni2p of the samples can split into two peaks in 2p_3/2_ after fitting. One peak represents Ni^2+^ at around 854.2 eV and the other is Ni^3+^ at around 855.4 eV, and a satellite peak presented around 861.0 eV. The overall binding energy of Ni2p_3/2_ exhibited a pattern of rising and then falling with the increase in sintering temperatures (NCM750, NCM800, and NCM850 values were 854.4 eV, 854.6 eV, and 854.4 eV, respectively), indicating the variation in valance states of Ni. The higher binding energy of Ni reflects a tendency toward an oxidation state. Furthermore, the relative contents among different valance state elements can be reflected using the ratio of peak areas [44]. In Figure 4b, the highest area ratio among the three samples for Ni^3+^/Ni^2+^ in NCM800 can be observed. Due to the fact that the phenomenon of cation mixing in NCM layered oxide occurs on the sites of Ni^2+^ and Li^+^, a higher Ni^3+^ proportion can reduce the degree of cation mixing in the crystal structure, which is consistent with the XRD results in Table 1. Figure 4d–f shows that the two contributions in O1s at 529.0 eV and 531.2 eV were active oxygen (O_active_) peaks and lattice oxygen (O_lattice_) peaks. O_lattice_ resulted from metal–oxygen bonds in the TM layers within the NCM structures, whereas O_active_ mainly included active oxygen species (Li_2_CO_3_/LiOH) and surface adsorbed species (OH^−^ or H_2_O) with a high binding energy [45]. NCM800 displayed the highest value of O_lattice_/ (O_active_ + O_lattice_) among all of the samples (NCM750, NCM800, and NCM850 values were 41.9%, 45.7%, and 44.5%, respectively), implying a more stable structure with fewer surface impurities. These results may lead to improved electrochemical properties for NCM800.

**Figure 5:**
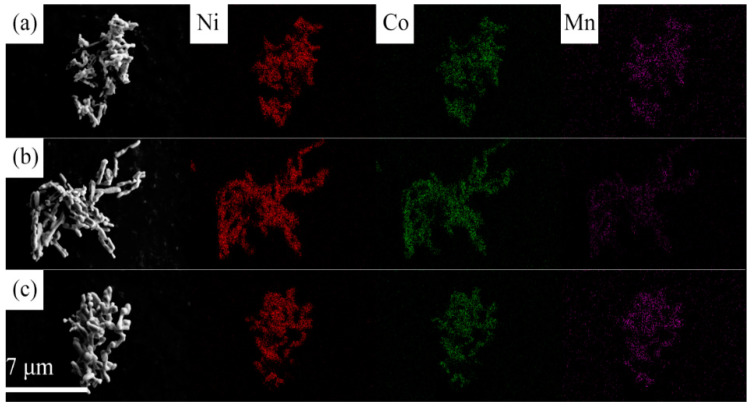
EDX mapping images of (**a**) NCM750, (**b**) NCM800, and (**c**) NCM850.

**Figure 6:**
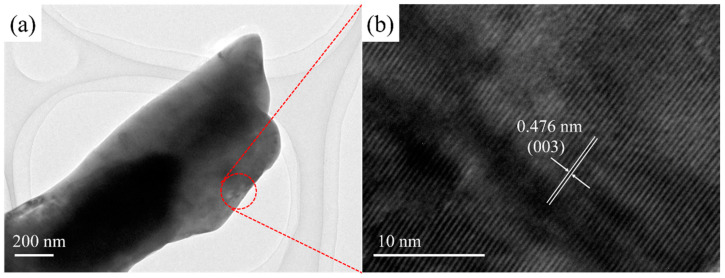
(**a**) TEM and (**b**) HRTEM images of NCM800.

SEM-EDX mapping diagrams are shown in Figure 5. The elements of Ni (red), Co (green), and Mn (purple) present uniform distributions on the surface of micron-sized monodisperse particles. Then, the atomic ratios of Ni:CO:Mn in the three samples were tested using SEM-EDX, as shown in Table S1. The data indicate that NCM800 is a stoichiometric sample, and there was a minor loss of Ni in NCM750 and NCM850. To further investigate the internal morphology of NCM800, TEM and high-resolution TEM images are shown in Figure 6. Figure 6a shows a low-magnification TEM image of NCM800, and Figure 6b shows a high-resolution image of the circled area in Figure 6a. Parallel lattice stripes can be observed in Figure 6b, and the width of the inter-planar spacing is 0.476 nm, which corresponds well with the (003) planes of the NCM structure. In addition, in the selected area electron diffraction (SAED) patterns are shown in Figure S2, a clear array of electron diffraction patterns rather than Debye rings can be observed, which confirms the single crystal properties of NCM800.

**Figure 7:**
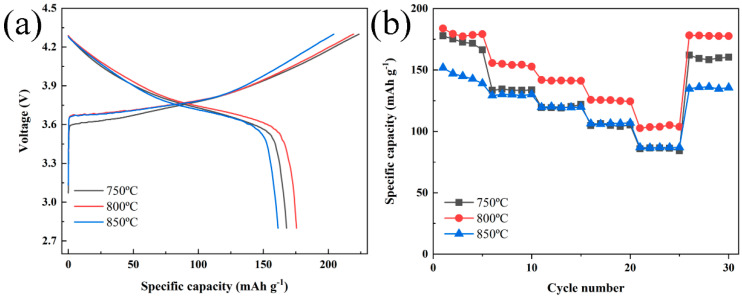
(**a**) The initial charge–discharge curves at 0.1 C, and (**b**) rate performance of NCM622 at different sintered temperatures.

Figure 7a shows the initial charge–discharge curves of samples with different sintering temperatures in a voltage window of 2.8–4.3 V at 0.1 C. The initial discharge capacities were 167.9, 175.5, and 162.6 mAh g^−1^, with Coulombic efficiencies of 75.1%, 80.3%, and 79.0% for the samples of NCM750, NCM800, and NCM850, respectively. NCM800 presented the highest discharge capacity, mid-value voltage, and Coulombic efficiency, indicating a higher energy density, smaller polarization, and irreversible capacity for the materials. Most irreversible capacity loss was ascribed to cation mixing, which is similar to the analysis of Table 1. The rate capabilities of the materials were tested at 0.1 C, 0.5 C, 1 C, 2 C, 5 C, and then at 0.1 C again, with each rate applied for five cycles. NCM800 had a better rate performance than NCM750 and NCM850, as shown in Figure 7b. At the rates of 0.1 C, 0.5 C, 1 C, 2 C, and 5 C, the initial discharge capacities could reach 183.8, 155.7, 141.9, 125.7, and 102.5 mAh g^−1^, respectively. The capacity retention of NCM800 was 93.0% with a capacity of 171.0 mAh g^−1^ after finally returning to 0.1 C, which was ascribed to the stable layered structure and lower polarization during the variation of current densities.

**Figure 8:**
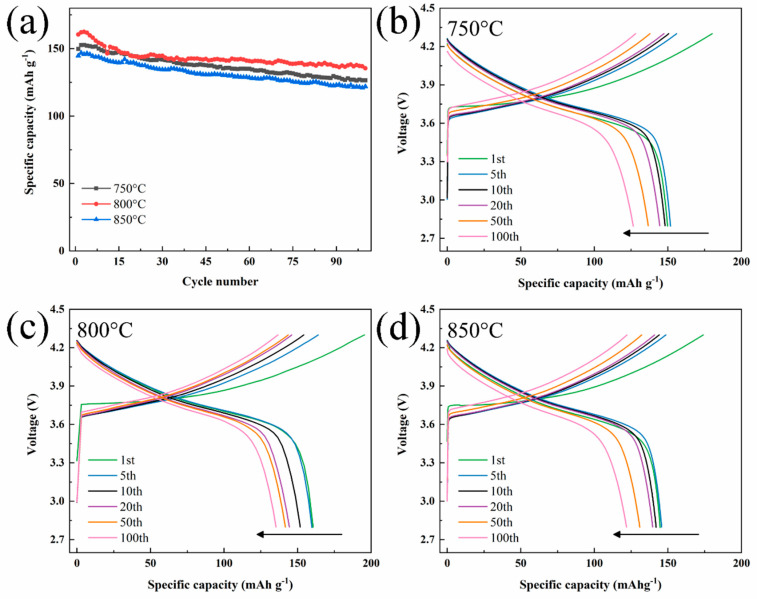
(**a**) The cycling performance of NCM622 at different sintering temperatures at 1 C for 100 cycles, and corresponding charge–discharge curves of (**b**) NCM750, (**c**) NCM800, and (**d**) NCM850 at 1 C for the 1st, 5th, 10th, 20th, 50th, and 100th cycles.

Figure 8 presents the cycling performance of NCM750, NCM800, and NCM850 at 1 C for 100 cycles. As seen in Figure 8a, the discharge capacities of NCM750, NCM800, and NCM850, respectively, delivered 149.7, 160.5, and 144.7 mAh g^−1^ from the 1st cycle to 126.4, 135.4, and 121.9 mAh g^−1^ in the 100th cycle. The three samples exhibited a similar trend of fading capacity, whereas the superior discharge capacity after 100 cycles of NCM800 was shown among all samples. The capacity difference is attributed to the relatively more monodisperse characteristics of the micron-sized particles of NCM800, which can mitigate side reactions between materials and electrolytes due to their stable structures and small specific surface areas. Figure 8b–d shows the charge–discharge curves of NCM750, NCM800, and NCM850, respectively, in the 1st, 5th, 10th, 20th, 50th, and 100th cycles. There is little difference in capacity retention among all the samples during the first few cycles. However, NCM800 presented a higher capacity retention from the 10th cycle until the end of cycling; this phenomenon can be explained by the fact that some inactive Li species, including inner impurities and residual Li on the surface, were formed under high-temperature calcination. These electronically insulated species gradually affected the capacity retentions during the middle and late cycling process, demonstrating that a suitable sintering temperature favors capacity retention upon cycling. 

Performance comparisons of NCM622 cathode materials under different preparation methods and types of particles are shown in Table 2. The traditional spherical NCMs could be obtained using the co-precipitation method, and the secondary particles were composed of randomly oriented nano-sized primary particles, which often present unstable cycle properties. Other methods can be used to synthesize primary particle NCM materials with monodisperse characteristics, and better electrochemical stabilities, because the larger size of the monodisperse particles could reduce the side reactions with electrolytes and inhibit the formation of crystal microcracks. The performance of NCM800 in this work can compete with that of other primary particle materials with monodisperse characteristics; however, our preparation method is more convenient than the sol–gel and molten salt methods.

**Figure 9:**
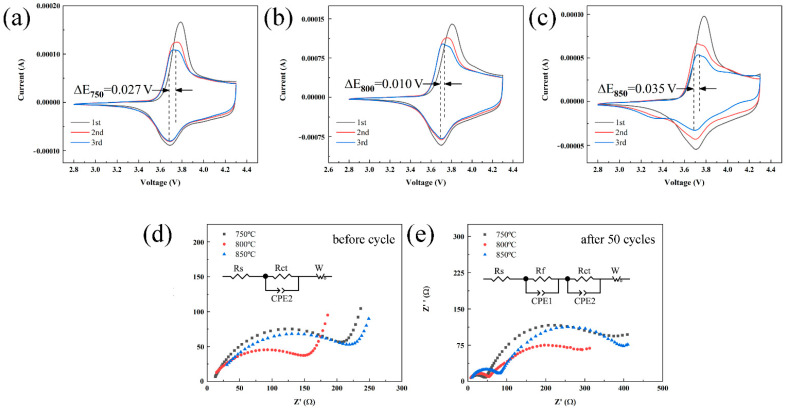
Cyclic voltammetry profiles of (**a**) NCM750, (**b**) NCM800, and (**c**) NCM850 in the 1st to 3rd cycle; Nyquist plots of NCM622 (**d**) before cycling and (**e**) after 50 cycles (the inset shows the equivalent circuit for fitting EIS results).

The CV curves of NCM750, NCM800, and NCM850 for the first three cycles are shown in Figure 9a–c. One pair of redox peaks around 3.6 V/3.8 V can be observed in all samples in the voltage range of 2.8–4.3 V with a scanning rate of 0.1 mV/s, which corresponds to the chemical redox reaction of Ni^2+^/Ni^4+^ [47]. The potential difference (ΔE) between the oxidation and reduction peaks implies the reversibility of the electrochemical reactions, and a smaller ΔE represents a lower polarization and higher reversible capacity. In addition, as shown in Figure 9a–c, NCM800 (0.010 V) shows a lower ΔE value than those of NCM750 (0.027 V) and NCM850 (0.035 V), which indicates that NCM800 has a reliable reversible capacity and the lowest polarization among the three samples.

In order to evaluate the electrochemical kinetic performance of the three samples, EIS results for NCM750, NCM800, and NCM850 before cycling and after 50 cycles are shown in Figure 9d,e. These plots are composed of semicircles at high to medium frequencies with slant lines at low frequencies. The equivalent circuits of the inset images are used to fit the curves. The high-frequency semicircles represent the ohmic resistance of electrolytes (R_s_) and the solid electrolyte interface film resistance (R_f_); the intermediate frequency semicircles are associated with charge–transfer resistance (R_ct_); the constant phase elements (CPE) are the surface film capacitance (CPE1) and the double-layer capacitance (CPE2); the low frequency slanted lines are related to the Warburg (W) diffusion process within the electrodes [48]. The corresponding fitting results are listed in Table S2. R_s_ values do not show significant changes before and after the cycling of samples. Note that only one set of semicircles corresponding to R_ct_ can be observed before cycling, the reason for the lack of R_f_ is that the stable formation of the solid electrolyte interface film needs to undergo an electrode activation process at the beginning of the cycle. The R_ct_ of values of NCM750, NCM850, and NCM850 increase from 198.8 Ω, 189.1 Ω, and 217.7 Ω to 380.6 Ω, 243.4 Ω, and 383.0 Ω, respectively, after 50 cycles. NCM800 shows relatively small changes of R_ct_, which indicates the rapid electrochemical interfacial transfer reaction between electrolytes and electrodes, and good conductivity during the cycling process.

## 4. Conclusions

Ni-rich LiNi_0.6_Co_0.2_Mn_0.2_O_2_ materials with micron-sized monodisperse particles were prepared using a simple oxalate solvothermal method combined with high-temperature calcination. The structure, morphology, and electrochemical properties of three cathode materials were systematically investigated, and the sintering temperature was found to be a significant factor for crystal growth and material morphology. The sample sintered at 800 °C exhibited micron-sized particles with monodisperse characteristics, and a stable layered structure. This material also delivered a high discharge specific capacity of 175.5 mAh g^−1^ with a Coulombic efficiency of 80.3% at 0.1 C. Its capacity retention was 84.4% after 100 cycles at 1 C, indicating a reliable cycle stability and a lower R_ct_ than those of other samples, implying a decreased ionic resistance during cycling. This study of NCM622 confirms that moderate micron-sized monodisperse particles can improve electrochemical performance. The current findings are expected to provide a facile approach to prepare cathode materials for LIBs with different scales of primary particles or complex components.

## Figures and Tables

**Table 1:** Lattice parameters of NCM622 samples with different sintering temperatures.

Temperature (°C)	a (Å)	c (Å)	c/a	I(003)/I(104)
750 °C	2.8772	14.2336	4.9470	1.61
800 °C	2.8702	14.2234	4.9555	1.75
850 °C	2.8791	14.2505	4.9496	1.41

**Table 2:** Comparison of electrochemical performance of NCM622 cathode materials in this work with those of previously reported research.

Method	Particle Size (μm)	Primary/ Secondary Particle	1st Capacity (mAh g^−1^)	Cycle Number	Capacity Retention (%)	Ref.
Solvothermal	2–3	Primary	175.5 (0.1 C)	100	84.4 (1 C)	This work
Carbonate co-precipitation	1.5–4	Secondary	152.0 (0.2 C)	100	81.5 (1 C)	[16]
Carbonate co-precipitation	3	Secondary	155.0 (0.1 C)	100	77.3 (1 C)	[46]
Hydroxide co-precipitation	1–2	Secondary	182.1 (0.1 C)	100	83.9 (1 C)	[44]
Oxalate co-precipitation	1	Secondary	174.3 (0.2 C)	100	93.2 (1 C)	[43]
Molten salt	1–3	Primary	183.0 (0.2 C)	300	94.0 (1 C)	[35]
Sol–gel	0.2	Primary	174.0 (0.1 C)	100	87.0 (1 C)	[47]

## Data Availability

The data presented in this study are available on request from the corresponding author.

## Supplementary Materials

The following are available online at https://www.mdpi.com/article/10.3390/ma14102576/s1, Figure S1: XRD patterns of NCCO precursor samples, Figure S2: SAED patterns of NCM800, Table S1: The atomic ratios of NCM622 tested by SEM-EDX, Table S2: EIS fitting results of the equivalent circuit of NCM622.

## References

[1] Goodenough J.B. (2014). Electrochemical energy storage in a sustainable modern society. Energy Environ. Sci..

[2] Tarascon J.-M., Armand M. (2001). Issues and challenges facing rechargeable lithium batteries. Nat. Cell Biol..

[3] Bianchini M., Roca-Ayats M., Hartmann P., Brezesinski T., Janek J. (2019). Hin und zurück–die Entwicklung von LiNiO_2_ als Kathodenaktivmaterial. Angew. Chem..

[4] Manthiram A. (2011). Materials Challenges and Opportunities of Lithium Ion Batteries. J. Phys. Chem. Lett..

[5] Shetti N.P., Dias S., Reddy K.R. (2019). Nanostructured organic and inorganic materials for Li-ion batteries: A review. Mater. Sci. Semicond. Process..

[6] Ding Y., Mu D., Wu B., Wang R., Zhao Z., Wu F. (2017). Recent progresses on nickel-rich layered oxide positive electrode materials used in lithium-ion batteries for electric vehicles. Appl. Energy.

[7] Xia Y., Zheng J., Wang C., Gu M. (2018). Designing principle for Ni-rich cathode materials with high energy density for practical applications. Nano Energy.

[8] Myung S.-T., Maglia F., Park K.-J., Yoon C.S., Lamp P., Kim S.-J., Sun Y.-K. (2017). Nickel-Rich Layered Cathode Materials for Automotive Lithium-Ion Batteries: Achievements and Perspectives. ACS Energy Lett..

[9] Xu J., Lin F., Doeff M., Tong W. (2016). A review of Ni-based layered oxides for rechargeable Li-ion batteries. J. Mater. Chem. A.

[10] Zhang S., Ma J., Hu Z., Cui G., Chen L. (2019). Identifying and Addressing Critical Challenges of High-Voltage Layered Ternary Oxide Cathode Materials. Chem. Mater..

[11] Noh H.-J., Youn S., Yoon C.S., Sun Y.-K. (2013). Comparison of the structural and electrochemical properties of layered Li[Ni_x_Co_y_Mn_z_]O_2_ (x = 1/3, 0.5, 0.6, 0.7, 0.8 and 0.85) cathode material for lithium-ion batteries. J. Power Sources.

[12] Jung S.-K., Gwon H., Hong J., Park K.-Y., Seo D.-H., Kim H., Hyun J., Yang W., Kang K. (2014). Understanding the Degradation Mechanisms of LiNi_0.5_Co_0.2_Mn_0.3_O_2_ Cathode Material in Lithium Ion Batteries. Adv. Energy Mater..

[13] Fang R., Miao C., Nie Y., Wang D., Xiao W., Xu M., Wang C. (2020). Degradation mechanism and performance enhancement strategies of LiNi_x_CoyAl1_−x−y_O_2_ (x ≥ 0.8) cathodes for rechargeable lithium-ion batteries: A review. Ionics.

[14] Sari H.M.K., Li X. (2019). Controllable Cathode–Electrolyte Interface of Li[Ni_0.8_Co_0.1_Mn_0.1_]O_2_ for Lithium Ion Batteries: A Review. Adv. Energy Mater..

[15] Liu W., Oh P., Liu X., Lee M.-J., Cho W., Chae S., Kim Y., Cho J. (2015). Nickel-reiche Lithium-Übergangsmetall-Schichtverbindungen für Hochenergie-Lithiumionenakkumulatoren. Angew. Chem..

[16] Ma Y., Li L., Wang L., Luo R., Xu S., Wu F., Chen R. (2019). Effect of metal ion concentration in precursor solution on structure and electrochemical performance of LiNi_0.6_Co_0.2_Mn_0.2_O_2_. J. Alloys Compd..

[17] Zheng J., Yan P., Estevez L., Wang C., Zhang J.-G. (2018). Effect of calcination temperature on the electrochemical properties of nickel-rich LiNi_0.76_Mn_0.14_Co_0.10_O_2_ cathodes for lithium-ion batteries. Nano Energy.

[18] Ren D., Shen Y., Yang Y., Shen L., Levin B.D.A., Yu Y., Muller D.A., Abruña H.D. (2017). Systematic Optimization of Battery Materials: Key Parameter Optimization for the Scalable Synthesis of Uniform, High-Energy, and High Stability LiNi_0.6_Mn_0.2_Co_0.2_O_2_ Cathode Material for Lithium-Ion Batteries. ACS Appl. Mater. Interfaces.

[19] Ran Q., Zhao H., Hu Y., Shen Q., Liu W., Liu J., Shu X., Zhang M., Liu S., Tan M. (2018). Enhanced electrochemical performance of dual-conductive layers coated Ni-rich LiNi_0.6_Co_0.2_Mn_0.2_O_2_ cathode for Li-ion batteries at high cut-off voltage. Electrochim. Acta.

[20] Chen Z., Chao D., Lin J., Shen Z. (2017). Recent progress in surface coating of layered LiNi_x_Co_y_Mn_z_O_2_ for lithium-ion batteries. Mater. Res. Bull..

[21] Guo S., Yuan B., Zhao H., Hua D., Shen Y., Sun C., Chen T., Sun W., Wu J., Zheng B. (2019). Dual-component LixTiO_2_@silica functional coating in one layer for performance enhanced LiNi_0.6_Co_0.2_Mn_0.2_O_2_ cathode. Nano Energy.

[22] Lv Y., Cheng X., Qiang W., Huang B. (2020). Improved electrochemical performances of Ni-rich LiNi_0.83_Co_0.12_Mn_0.05_O_2_ by Mg-doping. J. Power Sources.

[23] He R., Wei A., Zhang L., Li W., Bai X., Liu Z. (2019). Studies on the electrochemical properties of nickel-rich Li_1.02_Ni_0.6_Co_0.2_Mn_0.2_O_2_ materials for lithium-ion batteries via cerium modifications. Solid State Ionics.

[24] Breuer O., Chakraborty A., Liu J., Kravchuk T., Burstein L., Grinblat J., Kauffman Y., Gladkih A., Nayak P.K., Tsubery M. (2018). Understanding the Role of Minor Molybdenum Doping in LiNi_0.5_Co_0.2_Mn_0.3_O_2_ Electrodes: From Structural and Surface Analyses and Theoretical Modeling to Practical Electrochemical Cells. ACS Appl. Mater. Interfaces.

[25] Duan J., Wu C., Cao Y., Huang D., Du K., Peng Z., Hu G. (2017). Enhanced compacting density and cycling performance of Ni-riched electrode via building mono dispersed micron scaled morphology. J. Alloys Compd..

[26] Liang R., Wu Z.-Y., Yang W.-M., Tang Z.-Q., Xiong G.-G., Cao Y.-C., Hu S.-R., Wang Z.-B. (2020). A simple one-step molten salt method for synthesis of micron-sized single primary particle LiNi_0.8_Co_0.1_Mn_0.1_O_2_ cathode material for lithium-ion batteries. Ionics.

[27] Zhang H., Yang S., Huang Y., Hou X. (2020). Synthesis of Non-spherical LiNi_0.88_Co_0.09_Al_0.03_O_2_ Cathode Material for Lithium-Ion Batteries. Energy Fuels.

[28] Langdon J., Manthiram A. (2021). A perspective on single-crystal layered oxide cathodes for lithium-ion batteries. Energy Storage Mater..

[29] Xu X., Huo H., Jian J., Wang L., Zhu H., Xu S., He X., Yin G., Du C., Sun X. (2019). Radially Oriented Single-Crystal Primary Nanosheets Enable Ultrahigh Rate and Cycling Properties of LiNi_0.8_Co_0.1_Mn_0.1_O_2_ Cathode Material for Lithium-Ion Batteries. Adv. Energy Mater..

[30] Fan X., Hu G., Zhang B., Ou X., Zhang J., Zhao W., Jia H., Zou L., Li P., Yang Y. (2020). Crack-free single-crystalline Ni-rich layered NCM cathode enable superior cycling performance of lithium-ion batteries. Nano Energy.

[31] Leng J., Wang J., Peng W., Tang Z., Xu S., Liu Y., Wang J. (2021). Highly-Dispersed Submicrometer Single-Crystal Nickel-Rich Layered Cathode: Spray Synthesis and Accelerated Lithium-Ion Transport. Small.

[32] Pang P., Tan X., Wang Z., Cai Z., Nan J., Xing Z., Li H. (2021). Crack-free single-crystal LiNi_0.83_Co_0.10_Mn_0.07_O_2_ as cycling/thermal stable cathode materials for high-voltage lithium-ion batteries. Electrochim. Acta.

[33] Zhang M., Shen J., Li J., Zhang D., Yan Y., Huang Y., Li Z. (2020). Effect of micron sized particle on the electrochemical properties of nickel-rich LiNi_0.8_Co_0.1_Mn_0.1_O_2_ cathode materials. Ceram. Int..

[34] Li F., Kong L., Sun Y., Jin Y., Hou P. (2018). Micron-sized monocrystalline LiNi_1/3_Co_1/3_Mn_1/3_O_2_ as high-volumetric-energy-density cathode for lithium-ion batteries. J. Mater. Chem. A.

[35] Qian G., Zhang Y., Li L., Zhang R., Xu J., Cheng Z., Xie S., Wang H., Rao Q., He Y. (2020). Single-crystal nickel-rich layered-oxide battery cathode materials: Synthesis, electrochemistry, and intra-granular fracture. Energy Storage Mater..

[36] Julien C.M., Mauger A. (2020). NCA, NCM811, and the Route to Ni-Richer Lithium-Ion Batteries. Energies.

[37] Wu H., Pang X., Bi J., Wang L., Li Z., Guo L., Liu H., Meng Q., Jiang H., Liu C. (2020). Cellulose nanofiber assisted hydrothermal synthesis of Ni-rich cathode materials with high binding particles for lithium-ion batteries. J. Alloys Compd..

[38] Shi Y., Zhang M., Fang C., Meng Y.S. (2018). Urea-based hydrothermal synthesis of LiNi_0.5_Co_0.2_Mn_0.3_O_2_ cathode material for Li-ion battery. J. Power Sources.

[39] Zuo Y., Xu G., Yin Q., Sun Y., Huang B., Liang G. (2020). Hydrothermal synthesized rugby–like LiNi_0.5_Co_0.2_Mn_0_.3O_2_ cathode materials with micro-nano structure for high performance Li-ion batteries. J. Electroanal. Chem..

[40] Zhang L., Wang H., Wang L., Cao Y. (2018). High electrochemical performance of hollow corn-like LiNi_0.8_Co_0.1_Mn_0.1_O_2_ cathode material for lithium-ion batteries. Appl. Surf. Sci..

[41] Cho T., Park S., Yoshio M., Hirai T., Hideshima Y. (2005). Effect of synthesis condition on the structural and electrochemical properties of Li[Ni_1/3_Mn_1/3_Co_1/3_]O_2_ prepared by carbonate co-precipitation method. J. Power Sources.

[42] Wang L., Wu B., Mu D., Liu X., Peng Y., Xu H., Liu Q., Gai L., Wu F. (2016). Single-crystal LiNi_0.6_Co_0.2_Mn_0.2_O_2_ as high performance cathode materials for Li-ion batteries. J. Alloys Compd..

[43] Yao X., Xu Z., Yao Z., Cheng W., Gao H., Zhao Q., Li J., Zhou A. (2019). Oxalate co-precipitation synthesis of LiNi_0.6_Co_0.2_Mn_0.2_O_2_ for low-cost and high-energy lithium-ion batteries. Mater. Today Commun..

[44] Huang Z., Wang Z., Guo H., Li X. (2016). Influence of Mg^2+^ doping on the structure and electrochemical performances of layered LiNi_0.6_Co_0.2_Mn_0.2_MgO_2_ cathode materials. J. Alloys Compd..

[45] Dai S., Yuan M., Wang L., Luo L., Chen Q., Xie T., Li Y., Yang Y. (2019). Ultrathin-Y_2_O_3_-coated LiNi_0.8_Co_0.1_Mn_0.1_O_2_ as cathode materials for Li-ion batteries: Synthesis, performance and reversibility. Ceram. Int..

[46] Zheng Z., Guo X.-D., Chou S.-L., Hua W.-B., Liu H.-K., Dou S.X., Yang X.-S. (2016). Uniform Ni-rich LiNi_0.6_Co_0.2_Mn_0.2_O_2_ Porous Microspheres: Facile Designed Synthesis and Their Improved Electrochemical Performance. Electrochim. Acta.

[47] Lee S.-W., Kim H., Kim M.-S., Youn H.-C., Kang K., Cho B.-W., Roh K.C., Kim K.-B. (2016). Improved electrochemical performance of LiNi_0.6_Co_0.2_Mn_0.2_O_2_ cathode material synthesized by citric acid assisted sol-gel method for lithium ion batteries. J. Power Sources.

[48] Zhu M., Li J., Liu Z., Wang L., Kang Y., Dang Z., Yan J., He X. (2021). Preparation and Electrochemical Properties of LiNi_2/3_Co_1/6_Mn_1/6_O_2_ Cathode Material for Lithium-Ion Batteries. Materials.

