# Supplementary material for: Micron-Sized Monodisperse Particle LiNi0.6Co0.2Mn0.2O2 Derived by Oxalate Solvothermal Process Combined with Calcination as Cathode Material for Lithium-Ion Batteries
Source: Materials (Basel). 2021 May 15;14(10):2576. doi: 10.3390/ma14102576 (PMC8155954; doi:10.3390/ma14102576)
Supplement: Supplementary file 1 [file materials-14-02576-s001.zip › materials-1204373-supplementary.pdf]

materials

MDPI

Supplementary Materials

Micron-Sized Monodisperse Particle $LiNi_{0.6}Co_{0.2}Mn_{0.2}O_2$ Derived by Oxalate Solvothermal Process Combined with Calcination as Cathode Material for Lithium-Ion Batteries

Zhuo Chen, Fangya Guo and Youxiang Zhang *

College of Chemistry and Molecular Sciences, Wuhan University, Wuhan 430072, China; chenzhuo0005@whu.edu.cn (Z.C); guofangya@whu.edu.cn (F.G.)
* Correspondence: yxzhang04@whu.edu.cn

Citation: Chen, Z.; Guo, F.; Zhang, Y. Micron-sized monodisperse particle $LiNi_{0.6}Co_{0.2}Mn_{0.2}O_2$ derived by oxalate solvothermal process combined with calcination as cathode material for lithium-ion batteries. *Materials* **2021**, *14*, x. https://doi.org/10.3390/xxxxx

Academic Editor: Carlos Fernandez

Received: 15 April 2021
Accepted: 13 May 2021
Published: May 2021

Publisher's Note: MDPI stays neutral with regard to jurisdictional claims in published maps and institutional affiliations.

Copyright: © 2021 by the authors. Licensee MDPI, Basel, Switzerland. This article is an open access article distributed under the terms and conditions of the Creative Commons Attribution (CC BY) license (http://creativecommons.org/licenses/by/4.0/).

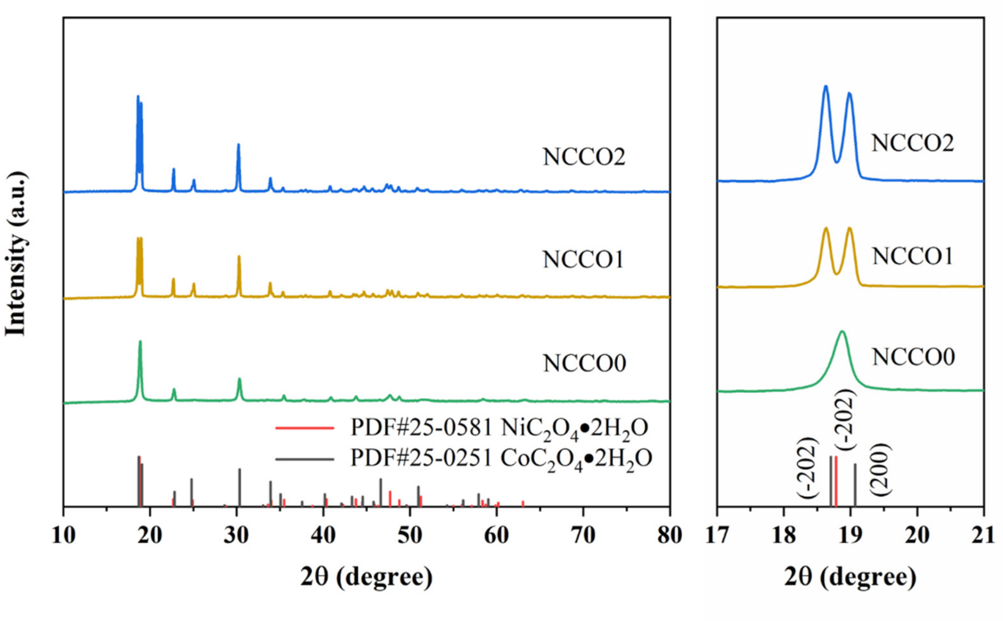

Figure S1. XRD patterns of NCCO precursor samples.

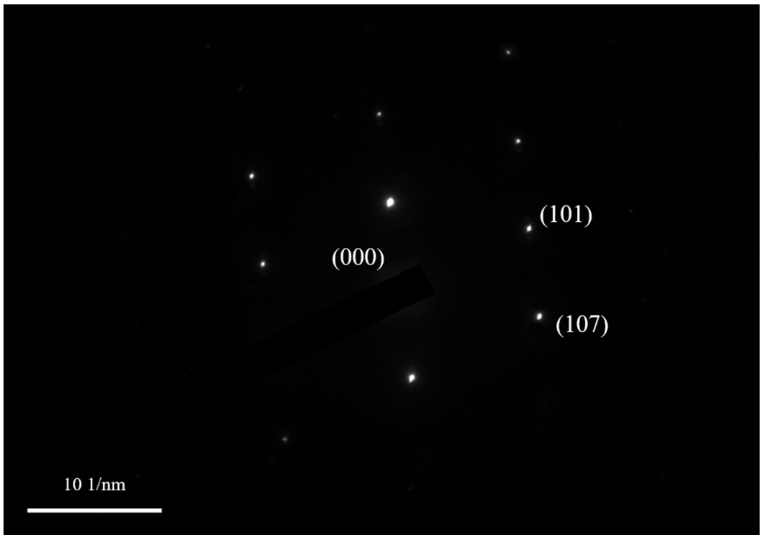

Figure S2. SAED patterns of NCM800.

Table S1 The atomic ratios of NCM622 tested by SEM-EDX.

Sample	Ni (%)	Co (%)	Mn (%)
NCM750	58.42	20.71	20.87
NCM800	59.21	20.28	20.51
NCM850	58.14	21.04	20.82

Table S2 EIS fitting results of the equivalent circuit of NCM622.

Samples	1st cycle		50th cycle		
	R_s (Ω)	R_{ct} (Ω)	R_s (Ω)	R_f (Ω)	R_{ct} (Ω)
NCM750	3.24	198.8	4.81	54.22	380.6
NCM800	1.92	189.1	2.83	54.38	217.7
NCM850	3.72	217.7	5.09	81.81	283.0
